# *CRABP-I* Expression Patterns in the Developing Chick Inner Ear

**DOI:** 10.3390/biology12010104

**Published:** 2023-01-10

**Authors:** Sheila Cardeña-Núñez, Antuca Callejas-Marín, Sergio Villa-Carballar, Lucía Rodríguez-Gallardo, Luis Óscar Sánchez-Guardado, Matías Hidalgo-Sánchez

**Affiliations:** Department of Cell Biology, School of Science, University of Extremadura, E06071 Badajoz, Spain

**Keywords:** retinoic acid, *Raldh3*, *Fgf10*, otic specification, endolymphatic apparatus, sensory patch

## Abstract

**Simple Summary:**

Retinoic acid is an important signal required for a high number of molecular and cellular processes in embryonic and postnatal development, as well as adult functions. Alterations of retinoic acid levels cause birth defects when it is deficient or in excess. *CRABP-I* governs the effects of retinoic acid sequestering and regulating its differential accumulation in the cytoplasm. The spatiotemporal expression patterns of *CRABP-I* in the developing chick inner ear were characterized to better clarify the potential role of *CRABP-I* in managing the retinoic acid signaling pathway. The heterogeneity of the *CRABP-I* expression pattern in the developing otic epithelium strongly suggests that *CRABP-I* may play a role in the specification of the dorsal-to-ventral axis of the otic anlagen, as well as in the specification of each sensory element as development proceeds. *CRABP-I* may also be involved in the final differentiation of neurons of the developing acoustic-vestibular ganglion that will innervate the sensory patches of this intricate sensory organ. These results suggest the direct involvement of *CRABP-I* in the epithelial specification and final differentiation of tissue and cells, as well as in morphogenetic events, which take place during embryonic development.

**Abstract:**

The vertebrate inner ear is a complex three-dimensional sensorial structure with auditory and vestibular functions, regarded as an excellent system for analyzing events that occur during development, such as patterning, morphogenesis, and cell specification. Retinoic acid (RA) is involved in all these development processes. Cellular retinoic acid-binding proteins (CRABPs) bind RA with high affinity, buffering cellular free RA concentrations and consequently regulating the activation of precise specification programs mediated by particular regulatory genes. In the otic vesicle, strong *CRABP-I* expression was detected in the otic wall’s dorsomedial aspect, where the endolymphatic apparatus develops, whereas this expression was lower in the ventrolateral aspect, where part of the auditory system forms. Thus, *CRABP-I* proteins may play a role in the specification of the dorsal-to-ventral and lateral-to-medial axe of the otic anlagen. Regarding the developing sensory patches, a process partly involving the subdivision of a ventromedial pro-sensory domain, the *CRABP-I* gene displayed different levels of expression in the presumptive territory of each sensory patch, which was maintained throughout development. *CRABP-I* was also relevant in the acoustic-vestibular ganglion and in the periotic mesenchyme. Therefore, *CRABP-I* could protect RA-sensitive cells in accordance with its dissimilar concentration in specific areas of the developing chick inner ear.

## 1. Introduction

The inner ear is an intricate sensory organ, regarded as an excellent model with which to study inductive and morphogenetic events, and the specification and determination of tissues and cells. Patterning events are initiated shortly after otic placode induction [1,2,3], perceptible through the generation of highly asymmetric and dynamic expression domains of specific regulatory genes, laying down the molecular blueprint for the consequent processes occurring during inner ear development. In this sense, understanding the molecular and cellular mechanisms regulating the patterning of the otic epithelium development is a main goal in the developmental biology field. This process is thought to be controlled by a network of diffusible morphogens through a multi-step mechanism [4,5,6,7,8,9,10,11,12,13,14,15,16].

Retinoic acid (RA), the active metabolite of retinol, is a small, nutritionally-derived molecule essential for normal embryonic and postnatal development, as well as for adult functions. However, it also causes birth defects when its presence is deficient or in excess. After its synthesis in a two-step process, involving firstly several alcohol dehydrogenases (ADHs) and retinol dehydrogenases (RDHs) and then retinaldehyde dehydrogenases (RALDHs), RA acts as an effective signaling molecule diffusing long distances through tissues. CYP1B1, a cytochrome P450 (CYP) enzyme, is also involved in RA synthesis during patterning events, catalyzing the conversion of retinol to retinaldehyde through a RALDH-independent pathway. In addition to RA synthesis, the degradation of RA by three cytochrome P450 (CYP) enzymes (CYP26A1, CYP26B1, and CYP26C1) plays a key role in the generation of RA concentration gradients. Functionally, RA is a ligand for two families of nuclear receptors, retinoic acid receptor (RAR) and retinoid X receptor (RXR), which bind to DNA sequences named retinoic acid response elements (RARE), activating the transcription of their downstream target genes. Thus, RA governs a wide variety of biological events, such as differentiation, proliferation, and apoptosis, as well as pattern formation during embryogenic and tumorigenic processes [17,18,19,20,21,22,23,24,25,26,27,28,29,30,31,32,33,34,35]

Several cellular retinoid binding proteins have been identified whose actions govern the effects of retinoids. RA binds with high specificity to cellular retinol-binding proteins (CRBPs), retinol-binding proteins (RBPs), and cellular retinoic acid binding proteins (CRABPs), solubilizing and stabilizing retinoids in aqueous media. In particular, CRABPs bind RA with high affinity and transport it to the nucleus to interact there with nuclear receptors (RAR/RXR). CRABPs would also sequester and regulate the differential accumulation of RA in the cytoplasm. In this sense, CRABPs buffer the free RA concentration in the cytoplasm, protecting RA-sensitive cells from overdose or regulating RA differential accumulation under conditions of vitamin A deficiency. In addition, CRABPs facilitate RA degradation by CYP26s. All these metabolic activities determine the final RA concentration in a specific tissue context to induce precise specification programs through the expression of many regulatory factors [17,22,36,37,38,39,40,41,42].

In vertebrates, there exist two highly conserved types of CRABPs: *CRABP-I* and *-II* [43]. In *Xenopus laevis*, an *xCRABP* gene has been identified [44,45], and in zebrafish, four *crabp* genes, designated *crabp1a*, *crabp1b*, *crabp2a*, and *crabp2b*, have also been characterized [46,47,48]. Tissue localization of CRABPs during embryogenesis and in the adult have provided ideas about the potential roles of these proteins in RA activity, especially in protecting RA-vulnerable tissues. In particular, *CRABP-I* expression has been observed in the mesenchymal cells of the limb bud, in various neural-crest-derived cells, in the developing nervous system and retina, in the branchial arches, in several craniofacial structures, in the lung, in the hair follicle dermal papilla, and in embryonic hearts, inter alia. Thus, the *CRABP-I* transcript distribution correlates well with structures known to be the targets of excess retinoid-induced teratogenesis: cells that express the *CRABP-I* gene cannot tolerate high levels of RA for their normal developmental functions [49,50,51,52,53,54,55,56,57,58,59,60]; for CRABP and *CRABP-II* proteins, see references therein). However, mouse *CRABP-I* and *-II* have been disrupted, singly or together, with no significant alteration in phenotype [61,62,63]. Only a forelimb alteration, a postaxial polydactyly, has been observed in mice homozygous for *CRABP-II* disruption [64].

In the developing mouse inner ear, *CRABP-I* expression has been observed in the otic placode at stage E8 [65]. Immunoreactions with anti-Crabp antibodies show that the otic vesicle is CRABP positive [66,67]. At 14.5 dpc, *CRABP-I* expression was observed outside of the otic capsule, probably corresponding to mesenchyme condensation, and in Kölliker’s organ [68]. Although western blot analysis showed that CRABP protein was present in the epithelium of the embryonic cochlea [69], *CRABP-I* mRNA was never detected in its sensory epithelium, the organ of Corti [68,70]. Loss-of-function studies, including the analysis of double *CRABP-I* and *CRABP-II* genes, do not reveal any structural alteration of the auditory structures, strongly suggesting that CRABPs could be dispensable for correct otic epithelial specification and morphogenesis [68]. In the developing chick inner ear, the presence of *CRABP-I* expression is observed primarily in the macula utriculi and in non-sensory regions flanking the macula sacculi [71]. To better elucidate the possible role of *CRABP-I* as a regulator of the RA signaling pathway, the main purpose of the present study was to characterize the spatiotemporal expression patterns of *CRABP-I* during the embryonic development of the chick’s inner ear. Thus, this analysis could provide insight into how the synthesis and action of RA are regulated and how this is linked to otic morphogenesis and to tissue and cellular specification processes.

## 2. Materials and Methods

### 2.1. Tissue Processing

We incubated fertilized White Leghorn chick eggs to obtain HH18, HH24, HH27, and HH32 chick embryos [72] in a humidified atmosphere at 38 °C. Chicken embryos were handled according to the protocol recommended by the European Union and the Spanish government concerning laboratory animals. Chicken embryos were fixed by immersion using 4% paraformaldehyde in 0.1M phosphate-buffered saline solution (PBS, pH 7.4) at 4 °C overnight. The next day, the embryos were rinsed with PBS 0.1M and cryoprotected overnight with a solution containing 10% sucrose in PBS. A sucrose solution with 10% of gelatin was used to embed the embryos before freezing for 1 min in an isopentane cooled to −70 °C. Then, chicken embryos were sectioned with a cryostat obtaining 20 μm thick sections. Twenty embryos were used per embryonic stage (n = 80).

### 2.2. In Situ Hybridization and Immunohistochemistry Staining Procedures

We performed in situ hybridization using the chick *CRABP-I* and *Fgf10* probes. The chick *CRABP-I* probe was obtained from the plasmid pBluescript-P, with *CRABP-I* full-length sequence (413 bp; NM_001030539.2), using HindIII and T7 enzymes to generate the antisense probe. The *Fgf10* probe helped to identify the developing sensory patches, as used previously [73]. All riboprobes were labeled with digoxigenin-11-UTP (Roche, Mannheim, Germany). In situ hybridization was performed according to Ferran et al. [74]. To obtain different levels of *CRABP-I* expression, we used several fixative conditions, different probe concentrations, and distinct incubation times with the chromogenic solution.

Immunohistochemistry was performed in some sections to detect acoustic-vestibular ganglionic neurons using a monoclonal 3A10 antibody (1:40; DSHB, AB_531874; [75,76,77]. Then, sections were incubated with a goat anti-mouse biotinylated secondary antibody (1:100; Sigma, St. Louis, MO, USA), followed by incubation with ExtrAvidin-biotin-horseradish peroxidase complex (1:200; Sigma). Next, peroxidase activity was revealed by adding a solution containing 0.03% diaminobenzidine (DAB) and 0.005% H_2_O_2_ to the sections. Finally, sections were mounted with Mowiol mounting solution.

### 2.3. Imaging

Images were acquired using 4×, 10×, or 20× objectives on a Zeiss Axiophot microscope. Images were analyzed with Adobe Photoshop software (version 22.2).

## 3. Results

### 3.1. CRABP-I Expression Pattern at the Otic Vesicle Stage (HH18-20)

**Figure 1:**
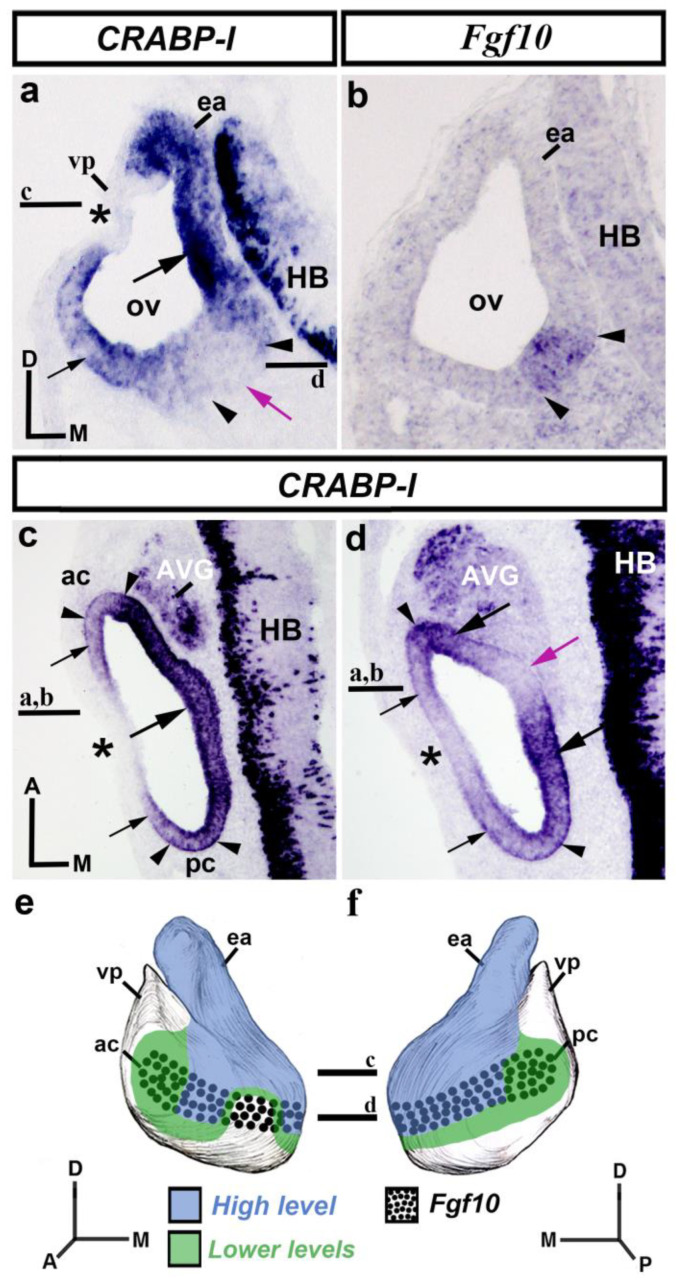
*CRABP-I* expression patterns at the otic vesicle stage, HH18-20. Transverse (**a**,**b**) and horizontal (**c**,**d**) sections treated with the *CRABP-I* probes. The patterns are heterogeneous, with higher levels in the entire dorsomedial portion (large black arrow in (**a**,**c**)) facing the hindbrain (HB; (**a**,**c**)), and including the growing endolymphatic apparatus (ea; (**a**)). Weaker levels of *CRABP-I* expression were observed in part of the lateral wall of the otic vesicle (small black arrows in (**a**,**c**,**d**)), including the anterior and posterior cristae (ac and pc; between arrowheads in (**c**)). A gap in expression was observed in the dorsolateral and lateral walls (asterisks in (**a**,**c**,**d**)). In the ventromedial aspect of the otocyst, there is an area without *CRABP-I* expression (purple arrows in (**a**,**d**)). The *Fgf10*-positive pro-sensory domain (between arrowheads in (**a**–**d**)) showed different levels of *CRABP-I* expression (ac and pc in (**c**); large black arrows in (**d**)) or non-expression (purple arrows in (**a**,**d**)). The AVG shows *CRABP-I* positive cells (**c**,**d**). (**e**,**f**), 3D *CRABP-I* expression pattern diagrams, showing anterior (**e**) and posterior (**f**) views of the otic vesicle. The horizontal sections were indicated. Dotted areas represent the pro-sensory *Fgf10*-positive domain. For the abbreviations, see the list. Orientation: A, anterior; D, dorsal; M, medial; P, posterior.

In transverse sections through the otic vesicle (HH18-20), high levels of *CRABP-I* expression were observed in its dorsomedial wall (large black arrow in Figure 1a), facing the hindbrain (HB; Figure 1a) and including the presumptive area of the incipient endolymphatic apparatus (ea; Figure 1a). In the dorsolateral otic epithelium, a small area was completely devoid of *CRABP-I* transcripts (asterisk in Figure 1a), associated in part with the presumptive domain of the vertical pouch (vp in Figure 1a) from which some semicircular canals will develop [75]. In the ventral aspect of the otic anlagen, evident *CRABP-I* expression was also detected in its lateral wall (small black arrow in Figure 1a). In some transverse rostral sections, a portion of the ventromedial pro-sensory area, *Fgf10*-positive (between arrowheads in Figure 1a,b [73]), was devoid of *CRABP-I* expression (purple arrow in Figure 1a; see also the horizontal section, purple arrow in Figure 1d).

To better study *CRABP-I* expression, horizontal sections treated with the *CRABP-I* probe were analyzed. In the dorsalmost sections (Figure 1c), strong *CRABP-I* expression was observed mostly in the vesicle’s medial wall (large arrow in Figure 1c) near the developing hindbrain (HB; Figure 1c). Weaker *CRABP-I* expressions, although still very evident, were observed in the anterior and posterior poles of the otic anlage, these areas including the presumptive domains of the anterior and posterior cristae (ac and pc; between arrowheads in Figure 1c). A small portion of the lateral wall of the otic vesicle was completely devoid of *CRABP-I* expression (asterisk in Figure 1c; see also asterisk in Figure 1a). In more ventral horizontal sections of the same chick embryo (Figure 1d), the otic epithelium showed heterogeneous expression of *CRABP-I*. Domains with stronger *CRABP-I* expression were clearly detected (large black arrows in Figure 1d). Two areas with weaker, although clearly manifest, levels of *CRABP-I* expression were also observed in the lateral portion of the otic epithelium (small black arrows in Figure 1d). In addition, *CRABP-I* expression was not detected in areas located in the lateral and medial walls of the otic vesicle (asterisk and purple arrow, respectively, in Figure 1d). That area present in the medial wall (purple arrow in Figure 1d; see also the purple arrow in Figure 1a) was within the pro-sensory domain (between arrowheads in Figure 1d) and would correspond to the presumptive domain of the macula sacculi [71]. The rostral and caudal contiguous areas with stronger *CRABP-I* staining, pointed to by large black arrows in Figure 1d, would correspond to the future macula utriculi and basilar papilla, respectively [77]. Furthermore, strong *CRABP-I* expression was also observed in the developing acoustic-vestibular ganglion (AVG in Figure 1c,d). Figure 1e,f summarize the *CRABP-I* expression pattern in the otic epithelium at stages HH18-20.

### 3.2. CRABP-I Expression Patterns at Stages HH24/25

**Figure 2:**
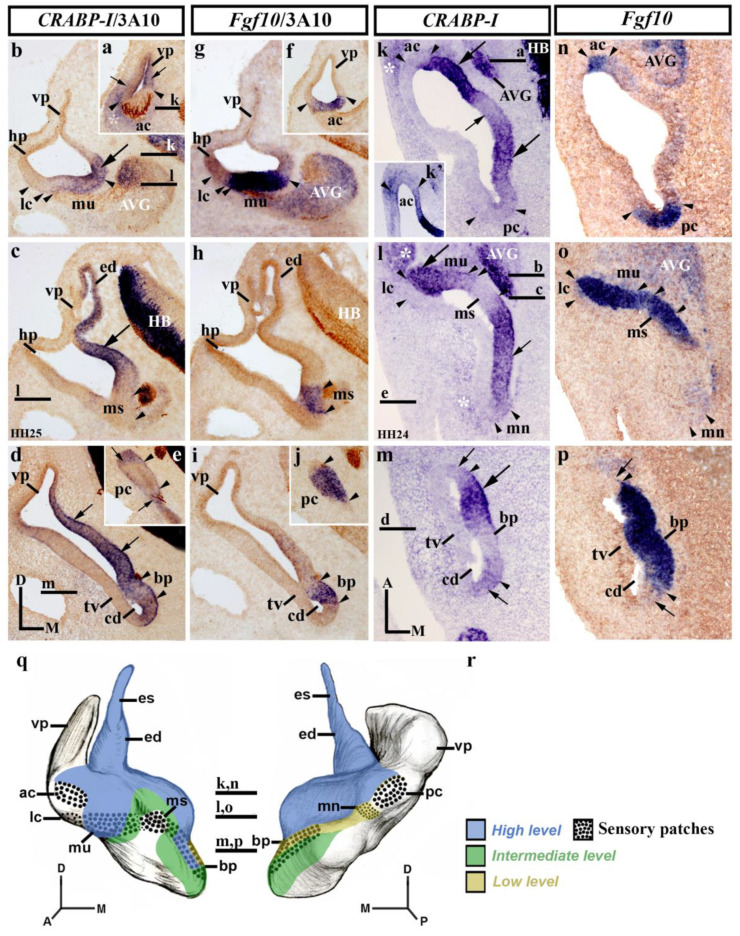
*CRABP-I* expression patterns at stage HH24/25. Transverse (**a**–**j**) and horizontal (**k**–**p**) sections through the inner ear anlagen and treated with the *CRABP-I* probes. The sensory patches were detected by the *Fgf10* probes (**f**–**j**,**n**-**p**). Otic innervation is identified by 3A10 immunoreaction in some sections (**a**–**j**). The presumptive areas of the developing sensory patches are indicated between arrowheads in all pictures. In a general view, *CRABP-I* expression was detected in the medial wall of stage HH24/25 otic anlagen (large and small arrows in (**b**–**d**), (**k**–**m**)). The developing endolymphatic duct was clearly included in the *CRABP-I*-labeling domain (ed in (**c**)). *CRABP-I* expression labeled a very small portion of the vertical pouch (vp) in the anterior and posterior aspects of the otic anlagen (small arrows in (**a**,**e**)), delimiting the anterior and posterior cristae, which were *CRABP-I* negative (ac and pc in (**a**,**e**,**k**,**k’**)). The lateral crista and the macula sacculi were also devoid of *CRABP-I* expression (lc and ms; (**b**,**c**,**l**)). The *CRABP-I* expressing domain included the macula utriculi (mu; (**b**,**l**)). The macula neglecta showed very low *CRABP-I* expression (mn; (**l**)). In the cochlear duct (cd), the *CRABP-I* expression was heterogeneous (arrows in (**d**,**m**)) and also in the basilar papilla (bp; between arrowheads in (**d**,**m**)). The presumptive domain of the tegmentum vasculosum was devoid of *CRABP-I* expression (tv in (**d**,**m**)). The acoustic-vestibular ganglion also showed *CRABP-I* expression (AVG in (**b**,**k**,**l**)). Some aspects of the underlying mesenchyme displayed evident *CRABP-I* expression (asterisks in (**a**,**k**,**l**)). (**q**,**r**), 3D *CRABP-I* expression pattern diagrams in anterior (**q**) and posterior (**r**) views of the stage HH24/25 inner ear. The horizontal sections are indicated. Dotted areas show the sensory patches. For the abbreviations, see the list. Orientation: A, anterior; D, dorsal; M, medial; P, posterior.

At stages HH24/25, the inner ear shows significant morphogenetic changes. The presumptive territories of almost all sensory patches are clearly recognized (Figure 2; see *Fgf10* expression [73]). In the rostralmost transverse sections (vp; Figure 2a,f), *CRABP-I* expression labeled the anterior wall of the vertical pouch (small arrows in Figure 2a), delimiting the innervated anterior crista (ac; between arrowheads in Figure 2a,f), which was apparently *CRABP-I* negative (Figure 2a). The observed *CRABP-I*-stained area of the otic epithelium corresponded to a small portion of the vertical pouch (vp in Figure 2a). In nearby caudalmost sections (Figure 2b,g), *CRABP-I* expression was observed in the medial wall (large arrow in Figure 2b), including the macula utriculi (mu in Figure 2b,g). At this level, the vertical and horizontal pouches (vp and hp), as well as the presumptive domain of the lateral crista (lc), were devoid of *CRABP-I* expression (Figure 2b,g). In adjacent caudal sections, the dorsomedial *CRABP-I*-labeling domain clearly included the developing endolymphatic duct (ed in Figure 2c,h). At this level, there was a decreasing dorsoventral gradient of *CRABP-I* expression, limiting dorsally the presumptive domain of the macula sacculi (ms; between arrowheads in Figure 2c,h), which was devoid of such expression. In the caudal portion of stage HH24/25 otic anlagen (Figure 2d,e,i,j), the *CRABP-I*-expressing domain observed in the medial otic epithelium (small arrows in Figure 2d) extended more ventrally in the medial wall of the cochlear duct (cd in Figure 2d). The presumptive territory of the basilar papilla showed evident, although weaker, *CRABP-I* expression (bp; between arrowheads in Figure 2d,i). The lateral wall of the cochlear duct, where the tegmentum vasculosum will develop, was clearly *CRABP-I* negative (tv in Figure 2d). In the most caudal transverse sections at the level of the posterior crista (pc; between arrowheads in Figure 2e,j), a strong *CRABP-I*-positive area bordered this *CRABP-I*-negative sensory patch (small arrows in Figure 2e).

In dorsal horizontal sections (Figure 2k,n), heterogeneous *CRABP-I* expression was clearly observed in the medial wall of stage HH24/25 otic anlagen (arrows in Figure 2k). At this level, the medial wall showed weaker *CRABP-I* expression at the insertion of the endolymphatic apparatus (small arrow in Figure 2k), bordered by areas of strong *CRABP-I* expression (large arrows in Figure 2k). Both the anterior and the posterior cristae (ac and pc; between arrowheads in Figure 2k,n) showed undetectable or very weak *CRABP-I* expressions (ac and pc in Figure 2k,k′). Some embryos showed very weak expression of *CRABP-I* in both cristae, indicating that, at the HH24/25 stage, a gradual loss of its expression is happening (not shown) with respect to the HH18/20 stage (Figure 1c). In the central portion of the vestibule (Figure 2l,o), the presumptive domain of the developing macula utriculi was clearly included in the *CRABP-I*-positive domain (mu; Figure 2l,o), with strong (lateral; large arrow in Figure 2l) and weaker (medial) levels of expression (Figure 2l). The lateral crista was clearly devoid of *CRABP-I* staining (lc; Figure 2l). One notes the sharp border of *CRABP-I* expression detected between the lateral crista and the macula utriculi. In the caudal aspect of this section, the *CRABP-I*-expressing area detected in the medial wall (small arrow in Figure 2l) extended caudally towards the incipient macula neglecta (mn in Figure 2l). One has to consider a very weak *CRABP-I* expression in this caudal sensory element (mn; Figure 2l). The macula sacculi were not labeled with *CRABP-I* probes (ms; Figure 2l,o; see also [71]). In more ventral horizontal sections through the developing cochlear duct (cd in Figure 2m,p), its wall showed a heterogeneous *CRABP-I* expression pattern (Figure 2m). The basilar papilla, located in the medial wall of the cochlear duct (bp; between arrowheads in Figure 2m,p), displayed different levels of *CRABP-I* expression. The lateral wall of the cochlear duct, including the presumptive area of the developing tegmentum vasculosum (tv; Figure 2m), was clearly *CRABP-I* negative (Figure 2m). As in the previous stage (HH18/20; Figure 1), *CRABP-I* expression was also observed in the acoustic-vestibular ganglion at this stage HH24/25 (AVG in Figure 2k,l; see also Figure 2b). In addition, some regions of the surrounding mesenchyme showed an evident *CRABP-I* expression (white asterisks in Figure 2k,l; see also Figure 2a). Figure 2q,r summarizes the *CRABP-I* expression pattern in the otic epithelium at stages HH24/25.

### 3.3. CRABP-I Expression Patterns at Stage HH27

**Figure 3:**
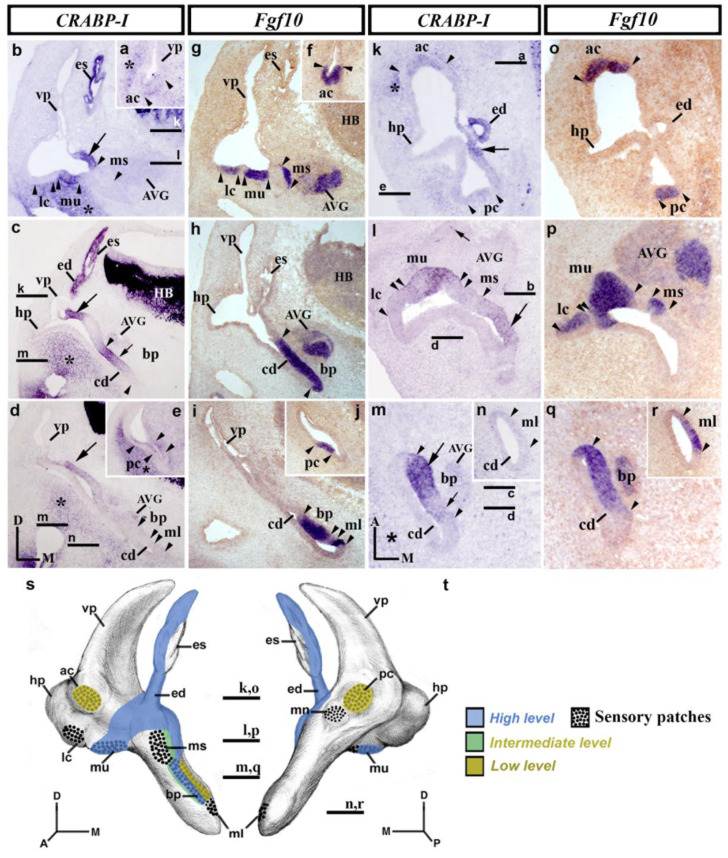
*CRABP-I* expression patterns at stage HH27. Transverse (**a**–**j**) and horizontal (**k**–**r**) sections through the inner ear anlagen treated with the *CRABP-I* (**a**–**e**,**k**–**n**) and *Fgf10* (**f**–**j**,**o**–**r**) probes. The presumptive areas of the developing sensory patches are indicated between arrowheads in all pictures. At this stage, the anterior and posterior cristae exhibited very weak *CRABP-I* labeling (ac and pc in (**a**,**e**,**k**)), whereas the lateral crista was *CRABP-I* negative (lc in **b**,**l**). The macula utriculi display *CRABP-I* expression (mu in (**b**,**l**)). The macula sacculi and macula neglecta were devoid of *CRABP-I* expression (ms in (**b**,**l**); mn, not shown). The wall of the endolymphatic apparatus (es and ed in **b**,**c**,**k**) and a portion of the vestibular wall at which it inserts (large arrow in (**b**-**d**,**k**)) were *CRABP-I* positive. In the developing cochlear duct (cd in (**c**,**d**,**h**,**i**,**m**,**n**,**q**,**r**)), *CRABP-I* expression was detected in its medial wall (small arrows in (**c**,**m**); large arrow in (**m**)), including the developing basilar papilla (bp in (**c**,**m**)). The macula lagena was *CRABP-I* negative (ml in (**d**,**n**)). At this stage, a few cells of the acoustic-vestibular ganglion were *CRABP-I* positive (AVG; small arrow in (**l**)). Some regions of the mesenchyme underlying the otic epithelium were *CRABP-I* stained (asterisks in (**a**–**e**,**k**,**m**)). (**s**,**t**), 3D *CRABP-I* expression pattern diagrams in anterior (**s**) and posterior (**t**) views of the stage HH27 inner ear. Dotted areas show the sensory patches. For the abbreviations, see the list. Orientation: A, anterior; D, dorsal; M, medial; P, posterior.

At stage HH27, the morphogenetic changes are more evident, and all the sensory epithelia are easily recognized [73]. Transverse sections through the rostralmost portion of the stage HH27 inner ear (Figure 3a–j) show the anterior crista to exhibit very light *CRABP-I* labeling (ac; between arrowheads in Figure 3a,f), while the contiguous otic wall now being devoid of *CRABP-I* expression (vp in Figure 3a). At stage HH27, the endolymphatic apparatus showed again strong *CRABP-I* labeling (es and ed in Figure 3b,c), except for a small area in the dorsomedial portion of the endolymphatic sac (Figure 3b). The wall of the vestibule where the endolymphatic apparatus inserts showed clear *CRABP-I* expression (large arrows in Figure 3b,c). At the same level, the macula utriculi displayed a manifest *CRABP-I* expression (mu in Figure 3b,g), whereas the lateral crista and the macula sacculi were completely devoid of such expression (lc and ms in Figure 3b,g). In the cochlear duct (Figure 3c,h,d,i), the *CRABP-I*-stained epithelium was associated with the basilar papilla (bp, between arrowheads in Figure 3c,h), with a small area of stronger *CRABP-I* expression (small arrow in Figure 3c). In the caudalmost transverse sections (Figure 3d,e,i,j), *CRABP-I*-labeled epithelium could only be detected weakly in a small area in the medial wall of the developing inner ear (large arrow in Figure 3d). In these caudal sections (Figure 3d,i), the macula lagena, which develops at the end of the cochlear duct, was devoid of *CRABP-I* expression (ml in Figure 3d,i). *CRABP-I* expression was very weak in the posterior crista (pc, between arrowheads in Figure 3e,j), the macula neglecta being *CRABP-I* negative (not shown). Furthermore, the underlying mesenchyme part of the membranous labyrinth presented different levels of *CRABP-I* expression staining (asterisks in Figure 3a–e).

Horizontal sections through the stage HH27 inner ear (Figure 3k–r) confirmed the above results. In the dorsal portion, the weak *CRABP-I* labeling observed in the anterior crista (ac; between arrowheads in Figure 3k,o) and its absence in the adjacent epithelium were also detected (Figure 3k; see also Figure 3a for a transverse section). One could consider the *CRABP-I* expression to be far weaker in the posterior crista (pc; between arrowheads in Figure 3k,o). In this section (Figure 3k,o), *CRABP-I* expression was observed in the endolymphatic duct (ed; Figure 3k; see also ed in Figure 3c) as well as in the area of the otic wall where the endolymphatic apparatus inserts into the wall of the vestibule (large arrow in Figure 3k; see also large arrows in Figure 3b–d). In the section through the central part of the vestibule (Figure 3l,p), the macula utriculi showed *CRABP-I* staining (mu in Figure 3l; see also Figure 3b). In the same sections, the *CRABP-I*-negative macula sacculi were bordered caudally by a *CRABP-I*-positive territory (large arrow in Figure 3l). The macula neglecta, located in the caudalmost portion of the otic epithelium, was devoid of *CRABP-I* expression (not shown). The mediocaudal *CRABP-I*-stained domain in the otic wall extended ventrally in the cochlear duct (cd in Figure 3m,q), labeling a dorsoventrally aligned band in its medial wall (arrows in Figure 3m), with two different levels of *CRABP-I* expression (compare the large and small arrows in Figure 3m). Note that the basilar papilla (bp; Figure 3m,q displayed a strong *CRABP-I* expression in its rostral part (large arrow in Figure 3m) but the weaker expression in its caudal part (small arrow in Figure 3m). The developing macula lagena was undoubtedly *CRABP-I* negative (ml; between arrowheads in Figure 3n,r; see also ml in Figure 3d,i, for transverse sections). At this developmental stage, and unlike the previously described stage (HH24/25), the acoustic-vestibular ganglion was almost completely devoid of *CRABP-I* expression (AVG in Figure 3b–d,l,m), except for a scattering of *CRABP-I*-positive cells in some sections (small arrow in Figure 3l). Some parts of the mesenchyme underlying the membranous labyrinth were also labeled by the expression of *CRABP-I* (asterisk in Figure 3k,m). Figure 3s,t summarize the *CRABP-I* expression pattern in the otic epithelium at stage HH27.

### 3.4. CRABP-I Expression Patterns at Stage HH32

**Figure 4:**
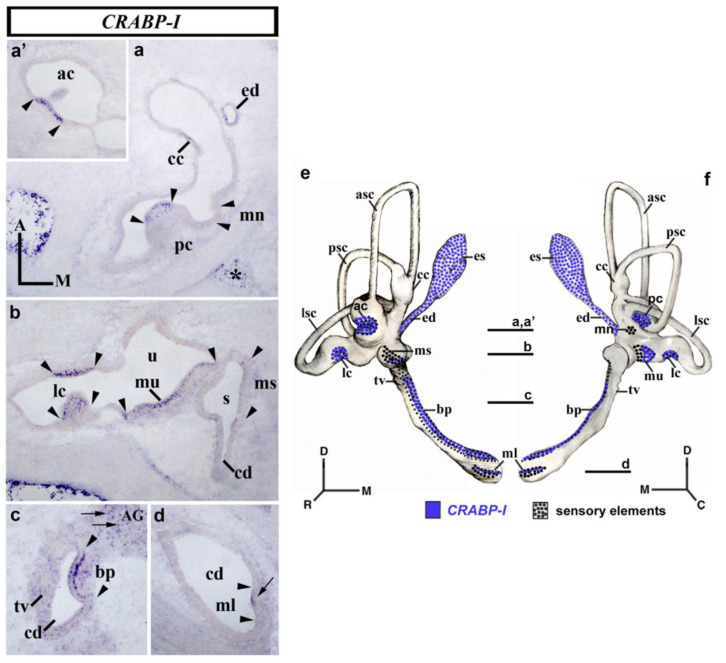
*CRABP-I* expression patterns at stage HH32. Horizontal sections through the inner ear anlagen treated with the *CRABP-I* probes. The sensory patches are indicated between arrowheads in all pictures. At this stage, all labeled cells identified in the otic epithelium exhibited high levels of *CRABP-I* expression. All the cristae displayed *CRABP-I*-expressing cells (pc, (**a**); ac, (**a’**); lc, (**b**)). The macula utriculi and the basilar papilla also showed *CRABP-I*-stained cells (mu in (**b**); pb in (**c**)). The macula sacculi and macula lagena showed a few *CRABP-I*-expressing cells (ms in (**b**); ml in (**d**)), although much less evident in the former. The macula neglecta was devoid of *CRABP-I* expression (mn, (**a**)). The endolymphatic duct and part of the crus commune exhibited *CRABP-I* expression (ed and cc; (**a**)). The AG and VG displayed scattered *CRABP-I*-positive cells (AG, small arrows in (**c**); VG, not shown). The mesenchyme far from the otic epithelium showed *CRABP-I* expression (asterisks in (**a**)). (**e**,**f**), 3D *CRABP-I* expression pattern diagrams, showing anterior (**e**) and posterior (**f**) views of the inner ear at stage HH34. The horizontal sections were indicated. Dotted areas represent the sensory domains. For the abbreviations, see the list. Orientation: A, anterior; D, dorsal; M, medial; P, posterior.

At day 8 of incubation (stage HH32; Figure 4), horizontal sections through the dorsal aspect of the inner ear showed that the anterior and posterior cristae displayed a strong *CRABP-I* expression in scattered cells (ac and pc; between arrowheads in Figure 4a,a’). At this stage, the macula neglecta, located in the proximity of the posterior crista, did not show any *CRABP-I*-positive cells (mn in Figure 4a). The endolymphatic apparatus (ed in Figure 4a) and a part of the crus commune (cc in Figure 4a) also showed cells expressing *CRABP-I*. In a horizontal section across the central part of the vestibule (Figure 4b), the lateral crista now displayed *CRABP-I*-labeled cells (lc; between arrowheads in Figure 4b). The whole of the macula utriculi showed more *CRABP-I*-stained cells, in particular in its lateral portion (mu in Figure 4b). The macula sacculi showed just a few *CRABP-I*-stained cells (not shown). In the horizontal sections through the cochlear duct (cd in Figure 4c,d), cells expressing the *CRABP-I* gene were detected in the basilar papilla (bp in Figure 4c). The macula lagena, located at the end of the cochlear duct, showed some *CRABP-I*-expressing cells in its apical border (ml; small arrow in Figure 4d). One notes that this *CRABP-I* positivity was observed in the apical portion of the sensory patches. As in the previously analyzed stages, the tegmentum vasculosum of the cochlear duct was *CRABP-I* negative (tv in Figure 4c). The acoustic and vestibular ganglia showed *CRABP-I*-stained cells (AG, small arrow in Figure 4c; VG, not shown). It is interesting to remark that, at this developmental stage, the mesenchyme just underlying the membranous labyrinth was completely devoid of *CRABP-I* expression. Nevertheless, there were some *CRABP-I*-stained cells in areas of the mesenchyme far from the otic epithelium, bordering the otic capsule (asterisk in Figure 4a). Figure 4e,f summarize the *CRABP-I* expression pattern in the otic epithelium at stage HH32.

## 4. Discussion

Retinoic acid (RA), an active metabolite of retinol that diffuses easily across cell membranes, regulates a wide range of biological processes, including inductive, specification, and morphogenetic events during development, as well as differentiation, proliferation, and apoptosis. Gradients of RA are generated from the source in its synthesis by means of RALDH and CYP1B1 enzymes. The dose-dependent effect of RA is also modulated by the activities of CRABP and CYP26. The final uneven distribution of RA may regulate the expression of spatially restricted transcription factors that have different thresholds for RA activation. For the CRABPs, they could provide additional negative feedback by preventing access of the ligand to receptors and facilitating RA degradation. Thus, the high conservation of their primary sequence, their spatiotemporal distributions during development, and pathological changes resulting from the ectopic expressions of CRABP clearly suggest that these proteins play a key role in tissue and cellular differentiation. While there are some clear differences between *CRABP-I* and *CRABP-II* [43,55,78,79], neither *CRABP-I^−/−^* or *CRABP-II^−/−^* single mutant nor *CRABP-I^−/−^/CRABP-II^−/−^* double mutant embryos show any abnormality under histological examination, suggesting that neither of these CRABPs is essential for normal development or even in adult life [43,61,62,63]. Therefore, *CRABP-I* and *CRABP-II* may share a functional redundancy, and some other alternate mechanisms might also act to regulate the RA signaling pathway. Although the role of CRABPs in embryonic development is still somewhat unclear, it is interesting to consider that tissues with greater sensitivity to RA teratogenicity display stronger levels of *CRABP* expression, suggesting that CRABPs may protect them against RA excesses [42].

### 4.1. Specification of the Chick Otic Vesicle by CRABP-I

A heterogeneous *CRABP-I* gene expression pattern was detected in the wall of the otic vesicle (this work; see also [71]). Higher levels were observed in its dorsomedial portion, an area facing the developing hindbrain and including the growing endolymphatic apparatus. The endolymphatic duct and sac continued to express the *CRABP-I* gene at stages HH24/27, with a salt-and-pepper pattern at stage HH32. At the otic vesicle stage, this strongly *CRABP-I*-expressing domain contains the *Raldh3*-expressing territory, with RALDH3 being the only retinaldehyde dehydrogenase involved in the synthesis of retinoic acid in the developing avian inner ear [80]. Thus, *CRABP-I* could modulate RA activity in the dorsomedial wall, regulating endolymphatic system development. Other regulatory factors could be considered in this molecular context. The *Gbx2* vertebrate homeobox gene is expressed in the early developing inner ear of vertebrates [81,82,83] in just the dorsomedial wall of the otocyst [84,85]. *Msx1* expression is an excellent marker of the endolymphatic apparatus, although it could also be involved in the differentiation of nearby vestibular structures, such as the semicircular canals and their associated cristae [86]. DAN, a BMP4 antagonist, is detected in the medial otic epithelium during avian inner ear development, and its altered expression generates endolymphatic duct and sac abnormalities [87]. Furthermore, a detailed study of the expression patterns of *Wnt*-related genes clearly suggests that a specific combination of these genes could be directly involved in otic epithelium regionalization. Concerning the specification of the dorsomedial domain, *Wnt2b* gene expression is detected exclusively in this area, whereas *Fzd8* expression labels the entire otic epithelium except for the dorsomedial domain [88].

As mentioned above, *Raldh3* expression starts in the most dorsomedial aspect of the otic epithelium at stage HH18. A few hours afterward (stage HH20), this *Raldh3*-expressing area extends ventrally, forming a dorsal-to-ventral expression gradient [80], stopping at the pro-sensory *Fgf10*-positive domain [73]. It is well known that *CRABP-I* acts by eliminating free RA in the cytoplasm to protect RA-sensitive cells, thus regulating the concentration and dose-dependent activity of RA [43]. Therefore, *CRABP-I* would modulate the RA activity involved in the specification of (i) the developing endolymphatic apparatus, which is subject to the effect of higher levels of *Raldh3* and *CRABP-I* expression (see above), (ii) the adjacent, vestibular, area of the otic anlagen, where the pro-sensory *Fgf10*-positive domain develops and this epithelial territory is probably exposed to lower RA concentrations, and different levels of *CRABP-I* expression (see below), and (iii) the ventralmost portion of the otocyst, where the cochlear duct will enlarge, and the otic epithelium showed weaker *CRABP-I* expression or non-expression. One could therefore hypothesize that, at the otic vesicle stage, the different levels of *CRABP-I* expression might be involved in the specification of otic elements along the dorsal-to-ventral axis of the otic anlagen. In this sense, the experiments in which RA was applied at the otic cup/vesicle stage showed that vestibular structures were more susceptible to the effects of RA than the cochlear duct. Of the three semicircular canals, the anterior was the most susceptible to RA treatment, while the crus commune was particularly resistant. The defect in the formation of the semicircular canal is related to the downregulation of early cell proliferation in the otocyst epithelium [89]. The molecular interactions with several other signaling pathways, such as FGFs, WNTs, BMPs, and SHH, would cooperate with an RA molecular mechanism in otic axis specification [2,90,91,92].

### 4.2. Specification of the Vestibular System by CRABP-I

An important aspect in the development of the vertebrate inner ear is the specification of the sensory elements within the otic epithelium. In birds, it occurs in a pro-sensory territory present in the ventromedial wall of the otic vesicle, which expresses the *Serrate1*, *Sox2*, and *Fgf10* genes [73,93]. In later stages of embryonic development, this pro-sensory band divides successively, resulting in six of the eight sensory elements—the lateral crista and the macula neglecta could be generated de novo by inductive mechanisms [73]. Our result showed that, at the otic vesicle stage, there was a heterogeneous *CRABP-I* expression pattern with respect to the pro-sensory ventromedial band. Stronger *CRABP-I* expression was detected in two separate areas within the pro-sensory territory, which probably correspond to the macula utriculi and basilar papilla primordia. Between these two areas, a smaller area devoid of *CRABP-I* expression was detected, probably corresponding to the presumptive macula sacculi. In addition, the two more strongly *CRABP-I* labeled parts are contiguous to areas with weaker (intermediate) levels of *CRABP-I* expression, which match well with the developing anterior and posterior cristae. This finding suggests that *CRABP-I* proteins could protect differently small areas aligned along the anterior-to-posterior *Fgf10*-expressing band against the effects of RA, thus governing their specification into different sensory elements.

At the next stage analyzed, HH24/25, the anterior and posterior cristae declined from their initial levels of *CRABP-I* expression towards no expression or levels undetectable by the in situ hybridization method. The already detectable macula neglecta displayed a very low level of *CRABP-I* expression, whereas the macula sacculi continued with a complete absence of *CRABP-I* transcripts. Nevertheless, the developing macula utriculi maintained very high levels of *CRABP-I* expression. The changes in *CRABP-I* expression could be due to variations in *Raldh3* expression, whose mRNA has been observed in areas contiguous to all cristae and the utricular and saccular maculae, even in scattered cells within the developing cristae [73]. At stage HH27, the dynamic expression pattern of the *CRABP-I* genes showed that the macula neglecta lost its low expression, as observed in the following stages, while the anterior and posterior cristae showed low expression compared with the absence shown in the previous stage (HH24/25). Expression in both cristae probably reflects a new role of *CRABP-I* in cell specification in each sensory patch as development proceeds. Thus, after stage HH27, the role that *CRABP-I* plays in the specification of sensory patches becomes progressively less relevant, with its expression being maintained in only a scattering of cells within those patches, as was observed at stage HH32. This suggests that the *CRABP-I* gene could play a significant part in the acquisition of final cell differentiation [44,94]. All these considerations would carry over to the *Cyp1B1* expression patterns previously described in chick inner ear development [95].

Other signaling pathways could be considered in this molecular context. With respect to the TGF-β superfamily, there is a mutual regulation between these developmental regulatory factors and *CRABP-I* expression [56,96,97]. In the developing inner ear, RA may suppress the transcription of *Bmp4*, a member of the BMP subfamily of TGF-β, blocking the development of semicircular channels at low doses, while sensory epithelia are apparently disturbed at higher doses [98]. To better study the possible role of *CRABP-I* in inner ear development, the ERK signaling mechanism should be considered [43].

### 4.3. Specification of the Basilar Papilla by CRABP-I

The auditory system of the inner ear develops from the ventral portion of the otic anlagen. The primordium of the basilar papilla, the auditory sensory patch, is located in the medial wall of the cochlear duct. In the mouse, western blot analysis in the cochlea shows that CRABP protein is present from E14.5 dpc to postnatal day 1 [69]. The study of the distribution of *CRABP-I* transcripts during embryogenesis confirms that its expression is restricted to Kölliker’s organ in two bands of expression, while no expression is detected in the neighboring organ of Corti [68] (see also [99] for CRABP). Several studies confirm the absence of *CRABP-I* expression in the adult sensory epithelia of mammals [68,69,70].

In our results, at the otic vesicle stage, the presumptive territory of the basilar papilla was labeled by high levels of *CRABP-I* expression. This area is located caudal to the macula sacculi, which was devoid of *CRABP-I* expression, and rostral to the posterior crista which presented lower expression. The different quantities of *CRABP-I* transcripts in these incipient sensory areas could be relevant to their early individual specifications mediated by RA activity, i.e., balancing the RA dosage (RALDH3 and CYP1B1 activities), sequestering (*CRABP-I* activity), and degradation (CYP26 activities). *CRABP-I* activity would also be significant as development progresses, as relative levels of *CRABP-I* expression are also observed at stages HH24 and HH27 in these three sensory patches (macula sacculi, basilar papilla, and posterior crista). In the late stage of development, HH27, it is notable that the already identifiable macula lagena, developed at the end of the cochlear duct, is *CRABP-I* negative. The macula lagena, which may originate from a subdivision of the presumptive area of the basilar papilla [3,73], could also be mediated by RA action. The specification of the macula lagena would then also be governed by the RA signaling pathway [80,95]; see [4] for the mouse inner ear).

Analysis in detail of the developing basilar papilla shows this auditory sensory element to present heterogeneous expressions of the *CRABP-I* gene. At stage HH24, three levels of *CRABP-I* expression were observed: higher levels in its rostralmost portion, intermediate levels in its caudalmost portion, and a small area between the two with lower or almost undetectable levels. At stage HH27, the basilar papilla displayed a rostral-to-caudal gradient of *CRABP-I* expression. These different levels of *CRABP-I* mRNA suggest that its rostral part is more sensitive to the effect of RA. In this sense, it is interesting to note that, at stage HH27, *Raldh3* is expressed in a narrow band in the caudalmost portion of the distal cochlear duct wall, not close to the basilar papilla. This dorsoventrally aligned *Raldh3*-expressing band appears contiguous and caudal to the auditory sensory element at stage HH32 [80]. Regarding the CYP1B1 activity, which also generates RA, the basilar papilla displays a rostral-to-caudal decreasing gradient of Cyp1B1 expression at stage HH32 [95]. This finding suggests that *CRABP-I* may protect the rostralmost portion of the basilar papilla from the RA produced by CYP1B1 activity rather than by the RALDH3 enzyme. These gradients might well be consistent with a cell differentiation gradient in the developing auditory patch.

### 4.4. CRABP-I and Otic Neurogenesis

RA has great relevance for neuronal differentiation and migration [100,101,102,103,104,105]. Otic neuroblast specification is a significant cell differentiation event in the developing vertebrate inner ear to form the acoustic and vestibular ganglia [8,93], whose neurons project their axons towards the developing otic epithelium to innervate all sensory elements following orderly connection patterns [106,107]. At the otic vesicle stage, the neurogenetic area has been ascribed to the anteroventral wall [108], a domain defined by the expressions of such markers as *Neurog1* and *NeuroD/M* [2,15]. In the developing inner ear, the alteration of the RA signal reduces *NeuroD* expression in the anteromedial wall of the otocyst [109], an effect similar to that shown in hippocampus-derived neural stem cell cultures [110]. Our results showed strong *CRABP-I* expression in the presumptive area of the macula utriculi, where most otic neuroblasts generate [111]. The adjacent macula sacculi also participate in neuroblast generation [112]. In the chick inner ear, the developing macula sacculi were devoid of *CRABP-I* expression. Thus, different quantities of *CRABP-I*, and hence with different RA actions, could regulate the origin of sub-populations of otic neuroblasts. This finding could fit well with a temporal link between vestibular/acoustic neuronal and utricular/saccular macular fate specifications [71]. The possible role of *CRABP-I* in otic neurogenesis would also be in line with its suggested involvement in the differentiation and migration of neurons in the developing chick optic tectum [54]. Moreover, *CRABP-I* could be a candidate for mediating mesenchyme-to-epithelium transitions [113], a key event in otic neurogenesis [108]. Experimental studies are needed to determine whether changes in *CRABP-I* expression are decisive for the differentiation and delamination of otic neuroblasts [67,114].

### 4.5. CRABP-I in Periotic Mesenchyme Development

Epithelial-mesenchymal tissue interaction is considered to be essential for normal inner ear formation, which would involve the RA signaling pathway [4,13,115,116]. In this sense, cellular retinoid binding proteins, in particular CRABPs, would play a major role, as has been shown in mouse organogenesis [99]. Our results showed *CRABP-I* expression in the periotic mesenchyme (see also [71], suggesting its involvement in the specification of discrete areas of this tissue and in the formation of the future otic capsule. Due to the proximity of these *CRABP-I*-expressing mesenchymal areas to the developing membranous labyrinth, *CRABP-I* proteins could clearly be involved in epithelial-mesenchymal interaction, regulating the RA activity in inner ear development. It is particularly noteworthy that, in the mouse embryonic inner ear, all three *Raldhs* genes are expressed only in the otic epithelium, being excluded from the periotic mesenchyme [4], similar to the case observed for chick *Raldh3* expression [80]. However, in the zebrafish, *aldh1a2* expression is present in this latter tissue [117]. With respect to the avian inner ear, there is, therefore, no RA source present in the periotic mesenchyme other than that corresponding to CYP1B1 activity [95]. Thus, strong *Cyp1B1* expression was detected between the otic epithelium and the hindbrain at all the stages analyzed, particularly notable being the case of the mesenchyme near the wall of the cochlear duct. CYP26 and RARs, other members of the RA signaling pathway, may also play key roles in these interactive events (for a review, see [4]).

## 5. Conclusions

RA is an effective diffusible molecule produced by retinol metabolism that governs various biological events such as specification, differentiation, and morphogenesis during embryonic and postnatal stages, with affected areas being sensitive to different RA thresholds. *CRABP-I* proteins may be necessary to prevent the effects of RA; cells and tissues with increased sensitivity to RA show stronger levels of *CRABP-I* expression. The heterogenous *CRABP-I* expression observed in the developing otic epithelium is consistent with this hypothesis. Thus, *CRABP-I* proteins could regulate RA activity in the developing endolymphatic system, which has high levels of the enzyme RALDH3. Concerning the specification of the sensory patches, most of which develop from an anterior-to-posterior *Fgf10*-expressing band observed in the ventromedial otic vesicle, the different levels of *CRABP-I* expression along this pro-sensory domain could determine the precise location of each sensory element derived from it. Thus, the presumptive territories of the macula utriculi and the basilar papilla showed stronger *CRABP-I* expression, while the saccular macula, located between them, showed no *CRABP-I* expression. The anterior and posterior cristae, which develop at the ends of the *Fgf10*-positive band, expressed intermediate levels of *CRABP-I* expression at the otic vesicle stage and no *CRABP-I* expressions in the next stage under consideration. These differences could reflect different degrees of sensitivity to RA during their initial specification and final differentiation. Similar considerations could be taken into account during the development of the acoustic-vestibular ganglion and periotic mesenchyme.

## Data Availability

Data are contained within the article, and materials can be requested from the authors upon reasonable request.

## Abbreviations

ac	anterior crista
AG	acoustic ganglion
asc	anterior semicircular canal
AVG	acoustic-vestibular ganglion
bp	basilar papilla
cc	crus commune
cd	cochlear duct
*CRABP-I*	Cellular retinoic acid-binding protein I
ea	endolymphatic apparatus
ed	endolymphatic duct
es	endolymphatic sac
HB	hindbrain
hp	horizontal pouch
lc	lateral crista
lsc	lateral semicircular canal
ml	macula lagena
mn	macula neglecta
ms	macula sacculi
mu	macula utriculi
ov	otic vesicle
pc	posterior crista
psc	posterior semicircular canal
RA	Retinoic acid
s	saccule
tv	tegmentum vasculosum
u	utricle
vp	vertical pouch
